# Functional Antibody-Dependent Enhancement as an Immune Assessment Platform: Development, Standardization, and Translational Interpretation in Flavivirus Research

**DOI:** 10.3390/pathogens15050490

**Published:** 2026-05-01

**Authors:** Meng Ling Moi

**Affiliations:** 1 Department of Developmental Medical Sciences, School of International Health, Graduate School of Medicine, The University of Tokyo, Tokyo 113-0033, Japan; sherry@m.u-tokyo.ac.jp; Tel.: +81-3-5841-3515; 2 The University of Tokyo Pandemic Preparedness, Infection and Advanced Research Center (UTOPIA), Tokyo 108-0071, Japan

**Keywords:** antibody-dependent enhancement (ADE), flavivirus immunity, Fc gamma receptor (FcγR), functional immunoassays, immune assessment platform, vaccine evaluation, translational immunology, systems immunology, serological profiling, correlates of protection

## Abstract

Functional antibody-dependent enhancement (ADE) represents a fundamental and context-dependent characteristic of antiviral antibody responses, reflecting the dual capacity of antibodies to mediate both the neutralization and Fc receptor-dependent enhancement of infection. In flavivirus research, this duality complicates the interpretation of conventional serological metrics and limits the reliability of single-parameter correlates of immunity, particularly in populations with complex exposure histories. Over the past decade, functional ADE assays have evolved from specialized mechanistic tools into integrated immune assessment platforms supporting translational immunology, vaccine evaluation, and population-level immune surveillance. These platforms incorporate Fcγ receptor-relevant target cell systems, standardized viral inputs, dilution series-based profiling, quantitative enhancement metrics, and structured quality control frameworks to enable reproducible, comparable, and context-aware functional measurements across cohorts and laboratories. A central concept emerging from these developments is that ADE reflects a dynamic functional immune state rather than an intrinsic property of antibodies or a direct indicator of pathological risk. Accordingly, functional ADE platforms support the contextual interpretation of antibody activity across physiologically relevant conditions, facilitating discrimination between transient functional enhancement and clinically meaningful immunological risk. By integrating functional ADE metrics with serological, cellular, and epidemiological data, these platforms provide a structured framework for interpreting immune profiles in vaccine evaluation, booster strategy design, and population-level risk stratification. This review synthesizes the development, standardization, and global dissemination of functional ADE platforms and discusses key principles governing biological relevance, analytical robustness, and inter-site transferability. Emerging directions integrating functional ADE profiling with systems immunology, immunogenomics, and computational modeling are highlighted as pathways toward predictive, decision-support-oriented frameworks. By positioning ADE platforms as immune assessment infrastructures rather than isolated assays, this review underscores their value for mechanistic inquiry, translational interpretation, and preparedness-oriented responses to emerging viral threats in the absence of definitive correlates of protection.

## 1. Introduction

Flaviviruses—including the dengue virus (DENV), Zika virus (ZIKV), Japanese encephalitis virus (JEV), yellow fever virus (YFV), and West Nile virus (WNV)—continue to impose substantial and recurrent public health burdens across endemic regions, which is increasingly seen in areas experiencing vector expansion. Despite decades of progress in vaccinology, seroepidemiology, and clinical management, a persistent challenge in flavivirus immunology is that antibody quantity and binding breadth do not necessarily translate into protective function. In particular, the same antibody repertoire can mediate potent neutralization under one set of biological conditions yet facilitate enhanced infection under another. This functional ambiguity is a clinical challenge for the DENV, where sequential infections with heterologous serotypes and cross-reactive antibodies are associated with an increased risk of severe disease [1,2]. As a result, flavivirus vaccine development and evaluation require more than measurements of neutralizing antibody titers: they require functional platforms capable of interrogating Fc-dependent biology and the conditions under which antibodies shift from protective to potentially pathogenic activity. Notably, despite extensive research efforts, validated correlates of protection remain limited [1,2] for most flavivirus vaccines and natural infections, particularly in populations with complex immune histories. Functional assessment platforms, therefore, play a critical role in contextualizing serological measurements rather than serving as direct proxies for protection.

A central challenge in flavivirus immunology is that antibody responses do not map directly onto protective function. The same antibody repertoire can mediate neutralization under certain biological conditions while facilitating Fcγ receptor (FcγR)-dependent viral entry under others. This duality is particularly evident in dengue virus infection, where sequential exposure to heterologous serotypes and cross-reactive antibodies is associated with altered disease risk and immune modulation [1,2,3,4]. Sequential infection with antigenically related flaviviruses, including heterologous dengue serotypes or other flavivirus exposures, further contributes to variability in immune outcomes by shaping cross-reactive antibody repertoires and functional response profiles [5,6,7,8]. Antibody-dependent enhancement (ADE) should therefore be understood not as a singular phenomenon but as a context-dependent functional state arising from the interaction between antibody concentration, viral properties, and FcγR biology. In practical terms, antibody-mediated outcomes are inherently concentration-dependent, with neutralization typically observed at higher antibody levels, enhancement occurring at intermediate concentrations that favor optimal immune complex formation and FcγR engagement, and minimal effects at low concentrations [3,4,9,10,11]. Mechanistic studies have demonstrated that FcγR subtype, receptor density, and downstream signaling pathways critically influence immune complex uptake and infection outcomes [4,11,12,13,14,15,16,17]. These observations support a conceptual shift from viewing ADE as an intrinsic antibody property toward recognizing it as a dynamic and regulated immunological state [3,10,11,18].

This perspective has driven the development of functional ADE assay platforms that extend beyond single-readout measurements to incorporate defined cellular systems, standardized viral inputs, and quantitative evaluation frameworks [12,19,20,21,22]. Conventional neutralization assays, including PRNT and FRNT, remain essential for serological assessment but do not fully capture Fc-dependent functional activity, particularly in populations with complex immune histories [5,6,7,20,21,23]. Functional ADE platforms, therefore, provide a complementary analytical framework for interpreting antibody function in translational and epidemiological contexts.

Historically, flavivirus immunogenicity and risk assessment have relied heavily on neutralization assays, such as plaque reduction neutralization tests (PRNT) and focus reduction neutralization tests (FRNT). These remain indispensable for many purposes, including correlates of protection and serotyping. However, neutralization alone is not a sufficient surrogate for real-world function in populations with complex immune histories [5,6,7,20,21,23] (e.g., prior DENV infection, JEV vaccination, or exposure to related flaviviruses, as well as endemic vs. non-endemic settings). In addition, sequential exposure to antigenically related flaviviruses (superinfection), including heterologous dengue serotypes or viruses such as JEV and ZIKV, can further modulate disease outcomes by reshaping cross-reactive antibody repertoires and functional immune states [5,6,7,8]. In these settings, cross-reactive antibodies may partially neutralize, fail to neutralize, or enhance infection depending on the concentration, epitope specificity, and Fc-mediated engagement. Consequently, a parallel line of functional evaluation—explicitly incorporating FcγR biology—has become essential for both mechanistic research and translational decision-making, including the evaluation of vaccine candidates, booster concepts, monoclonal antibody strategies, and population-level immunity profiling.

Functional ADE assays have evolved substantially over the past decade, moving from proof-of-concept experiments to standardized and disseminated platforms used across diverse contexts. Several assay architectures are now commonly employed, including live-virus systems using FcγR-expressing cell lines by employing plaque assays, microneutralization tests, flowcytometry, reporter virus approaches, pseudotyped particles, and single-round infectious particles (SRIPs). Each has strengths and limitations. Live-virus FcγR-based assays can provide biologically direct readouts but require careful control of viral input, cell state, and biosafety constraints, and they remain the most faithful for the biological characterization of viruses and antibodies. Reporter and pseudovirus systems can improve throughput and standardization but may not fully recapitulate entry, replication, or innate signaling. SRIP-based systems offer a valuable middle ground for certain questions, enabling single-cycle infection readouts while reducing confounding by secondary rounds of replication; however, a full recapitulation of antibody interactions against a live virus, as well as limited access to detection reagents, may be a limiting factor [24,25]. Importantly, regardless of architecture, meaningful ADE assessment depends on disciplined platform design: defined target cell FcγR expression profiles; standardized virus preparation and quantification; pre-specified enhancement metrics; and an explicit plan for normalization across runs and consistent across laboratories.

A critical concept underlying platform-based functional ADE assays is that the “enhancement phenotype” is rarely captured by a single data point. Instead, functional profiles often exhibit concentration dependence and non-linear behavior, including bell-shaped curves and distinct peaks reflecting immune complex stoichiometry and receptor engagement thresholds depending on clinical samples. For translational purposes, this has led to the adoption of quantitative enhancement metrics that summarize the magnitude and/or area of enhancement across antibody dilutions, often expressed relative to virus-only controls and anchored to standardized viral inputs. When carefully implemented, such metrics enable a comparative evaluation across cohorts, vaccines, and time points, and they can be integrated with parallel immunological measurements (e.g., binding titers, neutralization titers, Fc effector functions, and cellular immunity) to provide a more coherent systems-level interpretation. The need for reproducible ADE functional platforms has become more urgent as the flavivirus field confronts increasingly complex questions. These include how pre-existing immunity from vaccination or prior infection shapes subsequent responses (pre-existing immunity); how booster strategies can reprogram immunity toward protection without increasing ADE risk; how cross-reactive antibody landscapes differ across age groups and geographic regions; and how to translate mechanistic insights into practical frameworks for vaccine safety and efficacy assessment. Addressing these questions requires assays that are not only biologically relevant but also robust and operationally transferable—capable of being implemented across multiple sites with consistent performance. Inter-laboratory transfer introduces additional variables (operator technique, reagent differences, instrument calibration, and local virus stocks), making platform-level standardization and quality assurance important.

**Figure 1:**
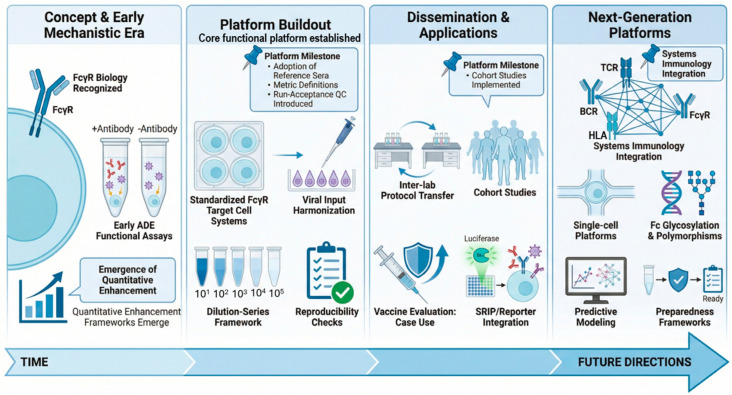
The evolution of functional ADE platforms from mechanistic studies to integrated systems frameworks. A schematic overview of the progressive development of functional antibody-dependent enhancement (ADE) platforms in flavivirus research. The establishment of FcγRIIa-expressing BHK platforms) [19,20,21,23] represented the first transferable ADE assessment system, and early investigations established the biological basis of Fcγ receptor (FcγR)-mediated viral uptake and enabled the development of initial functional ADE assays and quantitative enhancement frameworks. Subsequent platform buildout integrated standardized FcγR-relevant target cell systems, harmonized viral inputs, dilution series-based evaluation strategies, and reproducibility controls, leading to the establishment of core functional platforms. The dissemination and application phase was characterized by inter-laboratory protocol transfer, implementation in clinical cohort studies, and integration with reporter and single-round infectious particle (SRIP) systems for vaccine and translational evaluation. Emerging next-generation platforms incorporate systems immunology, single-cell profiling, Fc glycosylation and polymorphism analyses, predictive modeling, and preparedness-oriented frameworks. Together, these stages illustrate the transition from isolated mechanistic assays to transferable, multi-site functional infrastructures supporting translational and public health applications.

This review focuses on the development, standardization, and global application of functional antibody-dependent enhancement (ADE) assay platforms in flavivirus research. Rather than treating ADE assays as isolated protocols, they are considered here as integrated systems encompassing: (i) conceptual foundations in FcγR-dependent virology and immunology; (ii) platform development principles, including target cell selection, viral input standardization, and quantitative readout design; (iii) approaches to reproducibility and inter-laboratory dissemination; and (iv) applications across clinical cohorts, field studies, and vaccine evaluation pipelines (Figure 1). Complementary technologies, including reporter virus systems and emerging multi-parameter immunoprofiling approaches, are discussed as tools that can strengthen interpretability when integrated thoughtfully within a platform framework. Finally, next-generation directions that may transform functional ADE assays from retrospective measurements into predictive tools are outlined. These include integration with B cell receptor (BCR) repertoire analytics, single-cell immune profiling, epitope-resolved antibody mapping, Fc glycosylation, FcγR polymorphism-aware analysis, and computational modeling designed to map immunological breadth and depth to functional outcomes (Figure 1). In this forward-looking view, functional ADE platforms are positioned not merely as risk assessment tools, but as enabling infrastructure for rational vaccine design and pandemic preparedness—particularly in settings where pre-existing flavivirus immunity may shape responses to emerging pathogens or to vaccines deployed at scale. In summary, functional ADE assays have progressed from specialized experimental setups to widely used platforms that underpin a substantial fraction of modern flavivirus immunology and vaccinology. Continued progress will depend on how effectively these platforms are standardized, disseminated, and integrated with systems immunology to generate interpretable, comparable, and decision-relevant outputs linked to clinically meaningful outcomes. The purpose of this review is to consolidate practical platform principles and to provide a framework supporting rigorous mechanistic inquiry alongside translational application across diverse research and public health settings.

## 2. Conceptual Foundations of Functional ADE Platform Design

### 2.1. ADE as a Functional State, Not an Intrinsic Antibody Property

**Figure 2:**
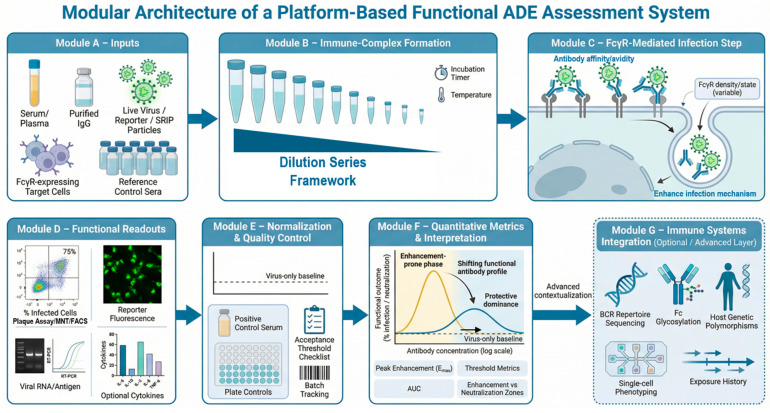
The end-to-end functional ADE platform pipeline for standardized evaluation and interpretation. Modular representation of an integrated functional ADE assessment platform, illustrating key analytical stages from experimental inputs to decision-relevant outputs. Module (**A**) defines core inputs, including serum or purified immunoglobulin, viral preparations (live virus, reporter systems, or SRIPs), FcγR-relevant target cells, and reference controls. Module (**B**) depicts immune complex formation across standardized dilution series under controlled incubation conditions. Module (**C**) represents FcγR-dependent uptake and entry, influenced by receptor density and cellular state. Module (**D**) summarizes primary functional readouts, including infection frequency, reporter activity, viral RNA or antigen levels, progeny production, and optional cytokine measurements. Module (**E**) outlines normalization and quality control procedures, incorporating virus-only baselines, positive control sera, plate controls, batch tracking, and acceptance thresholds to enable cross-site harmonization. Module (**F**) illustrates quantitative metrics and interpretive frameworks, including peak enhancement (E_max), area-under-curve (AUC) measures, threshold-based indices, and enhancement–neutralization zoning. Module (**G**) provides an optional advanced layer integrating immune repertoire, Fc glycosylation, host genetics, and single-cell data to support contextualized risk stratification and translational decision-making.

Antibody-dependent enhancement (ADE) is best conceptualized as a context-dependent functional state that emerges from the interaction of (i) antibody repertoire and concentration, (ii) viral antigenic landscape and particle properties, and (iii) Fcγ receptor (FcγR) biology in the target cell. In flavivirus systems, the same polyclonal serum can exhibit neutralization, no effect, or enhancement depending on dilution, target cell FcγR expression, and assay architecture. This indicates that “ADE” is not a single mechanistic outcome but rather a family of related phenotypes that share a common initiating event—the formation of virus–antibody immune complexes—followed by FcγR-dependent uptake, intracellular processing, and downstream readouts reflected in increased viral progeny [3,26]. Consequently, functional enhancement cannot be reliably inferred from binding assays, neutralization titers in the absence of FcγR, or rapid surrogate tests that bypass viral entry and intracellular replication. A meaningful assessment requires experimental systems that preserve Fc-dependent uptake and post-entry biology, as demonstrated in early FcγR-based platform studies [12,19,20,21,22,23,27,28], underscoring the necessity of infection-based platforms for mechanistic interpretation and translational decision-making. The relationships between immune complex formation, FcγR-mediated uptake, downstream functional readouts, and interpretive frameworks are summarized schematically in Figure 2.

### 2.2. Immune Complex Stoichiometry and Concentration Dependence

A core feature of ADE assays is non-linearity across antibody concentration, often manifesting as bell-shaped enhancement curves. At high antibody concentrations, virions may be neutralized or sterically blocked from productive entry; at intermediate concentrations, immune complexes can form at stoichiometries that optimize FcγR engagement and internalization; at very low concentrations, insufficient binding reduces FcγR-mediated uptake. Because this behavior is expected from first principles, platform design should treat dilution series as integral—not optional—and should predefine how enhancement will be summarized using peak-based, area-under-curve (AUC)-based, or threshold-based metrics. At the same time, near-undiluted or minimally diluted conditions may, in certain biological contexts, approximate in vivo antibody concentrations and immune complex configurations approximate in vivo antibody concentrations and immune complex configurations [5,22,27]. As illustrated in Figure 2B, antibody concentration governs the transition between neutralization-dominant and enhancement-prone states through immune complex stoichiometry. Hence, under these conditions, measured enhancement may reflect physiologically relevant functional states. Accordingly, undiluted or low-dilution data points can provide complementary information [22,23,27], particularly when interpreted within a structured dilution series framework. Comprehensive platform-based assessment, therefore, requires evaluation across the full concentration spectrum, enabling discrimination between transient, assay-dependent enhancement peaks and functionally meaningful profiles associated with natural infection, vaccination, or boosting.

### 2.3. Fcγ Receptor Engagement Determines Entry Route and Intracellular Fate

Fcγ receptor (FcγR) biology is not simply a “gateway” for viral entry; it influences intracellular routing, endosomal maturation, and downstream signaling pathways, which collectively determine whether immune complex uptake results in productive infection, abortive entry, or altered innate immune responses [13,14,15,29,30,31]. Figure 2C highlights how FcγR engagement and intracellular routing influence downstream functional outcomes following immune complex uptake. Key variables include receptor subtype (e.g., FcγRIIA vs. FcγRI/III), expression density, cell activation state, and receptor polymorphisms.

In practical assay terms, these variables motivate:

- The selection of FcγR-relevant target cells (cell lines or primary cells);
- The systematic documentation and monitoring of FcγR expression stability across passages;
- An avoidance of overgeneralization from single-cell models.

Experimental and molecular analyses have demonstrated that FcγRIIa-mediated uptake is coupled with distinct signaling pathways, including Syk-dependent and Src-family kinase cascades, which modulate viral replication efficiency, intracellular trafficking, and innate immune activation [12,14,15,16,17,32]. These pathways influence both entry efficiency and the post-entry cellular fate, shaping whether enhanced uptake translates into increased viral output or altered immune signaling. Importantly, FcγR-mediated enhancement—whether operating through entry-level (“extrinsic”) mechanisms or post-entry (“intrinsic”) modulation of cellular responses—does not, by itself, determine downstream clinical outcomes, as supported by mechanistic and cohort-based studies [8,10,13,16,18,30,31,33]. Functional ADE reflects localized, context-dependent cellular processes embedded within complex host–virus–immune interactions. Disease severity and protection emerge from the integration of viral burden, immune regulation, tissue tropism, and host genetic factors, together with that of FcγR-mediated uptake [34,35,36,37,38]. As such, FcγR engagement should be interpreted as one mechanistic layer within a broader immunological network. This reinforces the need for receptor-characterized target cell systems and for the integration of ADE readouts with multi-parameter immunoprofiling and clinical metadata in platform-based analyses.

### 2.4. Target Cell and Viral Input Context as Hidden Confounders

Even within a chosen target cell model, assay outcomes can vary substantially due to cell-cycle status, baseline interferon levels, differentiation state, and culture conditions and environment [28,39,40,41]. These factors modulate viral replication, innate restriction, and the magnitude of enhancement, and can introduce systematic bias if not appropriately controlled. For platform-level robustness, laboratories should standardize and document key parameters, including passage number windows, confluence thresholds at infection, incubation timing, and any stimuli (e.g., cytokines) that may alter FcγR expression or innate signaling. ADE readouts are influenced by viral input; however, within controlled and biologically relevant ranges, the overall enhancement patterns and concentration-dependent profiles are generally stable across biologically relevant input ranges [21,22,25]. Viral input itself is multidimensional, encompassing the genome copy number, infectious units, particle-to-PFU ratio, maturation state (e.g., prM cleavage), and aggregation, all of which can affect effective infectivity and antibody binding. Standardization, therefore, requires the selection of a primary input definition (e.g., PFU, FFU, MNT, or IU) and implementation of quality control procedures that protect against drift in infectious titer estimation, batch-to-batch particle heterogeneity, and storage-related loss of infectivity. Although moderate variation in viral input typically has a limited impact on qualitative ADE profiles, excessive divergence can influence cytopathic effects, replication kinetics, and downstream readouts. The coordinated overall control of viral input is therefore necessary to preserve biological interpretability and inter-study comparability.

### 2.5. Assay Architecture, Functional Endpoints, and Platform Logic

Different ADE assay formats interrogate distinct stages of the viral life cycle and therefore shape how “enhancement” is operationally defined. Live-virus replication assays capture entry, replication, and spread but may confound enhancement with downstream kinetics or multiple infection rounds. Single-cycle systems, including SRIP-based platforms, isolate early infection events and improve interpretability for specific mechanistic questions. While reporter and pseudotype systems enhance throughput and standardization, these assays may not fully recapitulate native flaviviral entry and replication biology. Target cell selection further influences functional readouts. Primary monocytes and macrophages provide physiological relevance but introduce donor variability, limited scalability, and technical complexity, and typically require higher multiplicities of infection to achieve reliable infection. Engineered or stable FcγR-expressing cell lines offer improved reproducibility and operational consistency but may represent simplified cellular contexts relative to primary myeloid cells. In this context, plaque-forming FcγR-expressing systems enable an integrated assessment of enhancement. As with all functional ADE platforms, performance remains dependent on viral stock quality, culture conditions, and assay standardization. Therefore, no single cellular model constitutes a universal “gold standard” for ADE assessment; rather, each captures distinct biological dimensions of Fc-dependent infection as demonstrated by comparative platform evaluations [13,16,28,39,40]. Ideally, functional readouts should not be interpreted in isolation but integrated with complementary immunological parameters, including neutralization profiles, antibody binding characteristics, cellular immunity, and exposure history, and where available, repertoire- and systems-level analyses such as BCR/TCR profiling and epitope mapping (Figure 2). Platform-based ADE assessment is most informative when embedded within such multi-parameter frameworks, enabling contextualized interpretation rather than reliance on enhancement measurements alone.

Accordingly, functional ADE platforms should predefine primary endpoints that align with study objectives. These may include the proportion of infected cells, reporter activity, viral RNA or antigen production, infectious progeny output, and, where relevant, cytokine or innate signaling markers. The endpoint selection should reflect whether the primary goal is vaccine safety profiling, mechanistic analysis of FcγR routing, or population-level immune mapping. A functional ADE “platform”, therefore, represents more than a protocol. It constitutes an integrated infrastructure comprising standardized workflows, defined inputs and controls, quantitative metrics, decision rules, and quality assurance thresholds. This platform logic enables multi-cohort and multi-site comparability and converts experimental measurements into interpretable, translationally relevant outputs that can inform vaccine evaluation and preparedness-oriented decision-making.

## 3. Application of Functional ADE Platforms Across a Research and Translational Context

### 3.1. Clinical, Cohort, and Field Applications

Platform-based functional ADE assays have been widely applied to characterize antibody-mediated responses in clinical cohorts and population-based studies [5]. In dengue-endemic settings, these platforms enable a systematic comparison of functional profiles across primary and secondary infections, age groups, and geographic regions. By integrating dilution series-based enhancement metrics with serological and clinical metadata, cohort studies can identify immune signatures associated with protection, subclinical infection, or increased disease risk [5,7,8,21,23,42,43]. Longitudinal sampling further enables the tracking of functional immune trajectories over time, including post-infection maturation, waning, and modulation following vaccination [44,45,46]. Such analyses provide insights into immune imprinting, pre-existing cross-serotype reactivity, and durability of functional responses that are not readily captured by conventional neutralization assays alone. During outbreak investigations and field-based surveillance, functional ADE platforms provide a mechanistic context for observed epidemiological patterns. When combined with viral genotyping, transmission dynamics, and seroprevalence data, ADE profiles inform hypotheses regarding disease severity, age-dependent risk, and regional variation in clinical outcomes [8,42,43]. Experience from multi-site studies demonstrates that harmonized platforms can generate comparable functional datasets even in resource-variable environments through modular assay design and standardized training protocols.

### 3.2. Vaccine, Booster, and Therapeutic Evaluation

Building on earlier FcγR-based platform development and quantitative enhancement frameworks [10,11,18,21], subsequent studies extended these systems to diverse translational applications, including vaccine evaluation and longitudinal immunity profiling [16,17,24,25,44]. Platform-based ADE assays have been applied to assess cross-genotype immunogenicity and concentration-dependent enhancement dynamics in flavivirus-naïve and pre-immune populations [6,47]. Importantly, FcγR-expressing cell-based functional assays have been cited in regulatory and policy-oriented evaluations of dengue vaccine immunogenicity [32,48,49,50].

The WHO ECBS technical review noted that conventional PRNT-based neutralization assays did not consistently discriminate between protective and non-protective cross-reactive antibody responses and that Fcγ receptor-bearing systems may offer improved discriminatory capacity [10,51] and subsequent validation studies [20,21,23,25,47]. This assessment reflects early platform-based work demonstrating the added interpretive value of FcγR-expressing systems in functional immune profiling [10,11,21]. Functional ADE platforms are also increasingly incorporated into monoclonal antibody and antibody-based therapeutic development. These systems enable a systematic evaluation of Fc-dependent effects alongside the neutralization potency and support assessment of Fc engineering and glycoengineering strategies for risk mitigation and regulatory submission.

### 3.3. Cross-Platform and Systems-Level Integration

To enhance interpretability, functional ADE platforms are frequently combined with complementary experimental systems, including plaque-based assays, reporter viruses, animal models, and ex vivo primary cell cultures. Cross-platform validation enables a dissection of early entry events, replication dynamics, and immunopathological consequences under controlled conditions. Large-scale applications enable the construction of immune landscape maps linking antibody repertoire features, exposure history, and functional outcomes. When integrated with BCR sequencing, epitope mapping, and single-cell profiling, these datasets support systems-level modeling of immune breadth, depth, and functional potential [7,8,42,43,52]. Such approaches facilitate the identification of recurrent response archetypes and inform stratification of populations according to functional risk profiles. Integrated use of multiple platforms strengthens mechanistic inference, reduces dependence on any single assay format, and supports the development of predictive frameworks for vaccine evaluation and surveillance.

### 3.4. Operational Deployment and Translational Value

Sustained deployment of functional ADE platforms requires coordinated operational frameworks. Key elements include centralized protocol repositories, standardized training modules, shared reference materials, and harmonized data management systems. Regular inter-laboratory benchmarking and joint quality assessment reviews support the continuous performance monitoring and early identification of systematic drift. Across clinical research, vaccine development, outbreak investigation, and systems immunology, platform-based approaches convert complex Fc-dependent biology into structured, comparable, and interpretable datasets. However, conclusions regarding assay sensitivity must distinguish between intrinsic cell-line biology and FcγRIIa expression fidelity, as reduced ADE detection in low-expression or unstable transfectants does not invalidate FcγRIIa-mediated platforms per se [38]. By linking mechanistic insight to operational scalability, functional ADE platforms bridge experimental immunology and public health practice. Their continued refinement and integration with emerging analytical technologies will further expand their utility in translational and preparedness-oriented research.

## 4. Methods and Reporting Standards for Platform-Based Functional ADE Assessment

### 4.1. Platform-Oriented Design and Experimental Architecture

**Figure 3:**
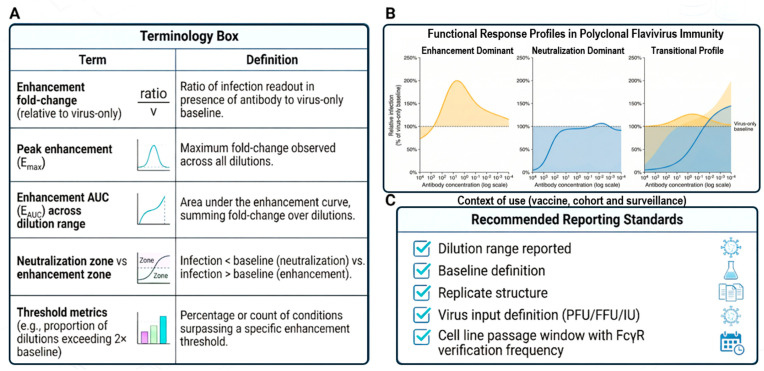
The standardized assay-metric framework for the consistent quantification and reporting of ADE. Integrated framework defining core quantitative metrics, response-profile classification, and reporting requirements to support reproducible and comparable ADE analyses across studies. (**A**) Terminology box defining the key quantitative metrics used in the functional ADE assessment. These include enhancement fold change relative to the virus-only baseline, peak enhancement (E_max), and enhancement area under the curve (E_AUC), which together capture both the magnitude and distribution of enhancement across dilution ranges. Neutralization and enhancement zones are defined relative to the baseline infection levels, while threshold-based metrics summarize the frequency or proportion of conditions exceeding the predefined enhancement criteria. These definitions establish a shared analytical language for cross-study comparison. (**B**) Representative functional response profiles across antibody dilution series in polyclonal flavivirus immunity. Profiles illustrate enhancement-dominant, neutralization-dominant, and transitional patterns, reflecting concentration-dependent shifts in functional activity. These patterns emphasize that ADE and neutralization represent continuous, context-dependent states rather than discrete categories, and they provide a basis for standardized response classification. (**C**) Context-aware reporting standards for vaccine evaluation, cohort studies, and surveillance applications. The key parameters include dilution range specification, baseline definition, replicate structure, viral input characterization (e.g., PFU/FFU/IU), and verification of FcγR-relevant target cell properties (e.g., expression stability across passages). Explicit reporting of these elements enables reproducibility, supports inter-laboratory harmonization, and ensures that quantitative metrics can be interpreted within their experimental and biological context. Together, this framework links assay design, quantitative readouts, and reporting conventions, enabling harmonized data interpretation and facilitating cross-platform and translational integration.

Functional ADE assays intended for comparative or translational use should be implemented within a platform-oriented design framework that integrates biological relevance, analytical reproducibility, and operational transferability. Rather than treating individual experiments as standalone measurements, platform-based assessment emphasizes standardized workflows, pre-specified performance criteria, and systematic documentation of key experimental variables. This approach enables a meaningful comparison across cohorts, time points, and laboratories. Core design elements include (i) defined antibody input formats (serum, plasma, or purified immunoglobulin), (ii) characterized viral preparations with stable infectivity profiles, (iii) FcγR-relevant target cell systems with monitored receptor expression, and (iv) reference materials that support longitudinal and cross-site normalization. Platform implementation should prioritize consistency of these elements across experimental cycles. The overall architecture of platform-based functional ADE assessment, including experimental inputs, analytical steps, and interpretive outputs, is outlined in Figure 3.

The selection and maintenance of target cell systems represent critical determinants of ADE assay behavior. Platforms should employ FcγR-expressing cells that reflect the intended biological context and exhibit stable receptor profiles across defined passage windows. Routine verification of FcγR expression levels is recommended, particularly following extended culture or culture in different environments. Cellular state variables, including confluence and, where feasible, metabolic status and baseline innate signaling signatures, should be monitored and controlled. When primary cells are used, donor variability and differentiation protocols should be documented to support a cross-study comparison. Different assay designs interrogate distinct stages of the viral life cycle and therefore shape how enhancement is operationally defined; as such, cross validations would be important. Study designs should pre-specify the biological scope of the selected system and avoid treating formats as interchangeable. To ensure that experimental architecture translates into interpretable and transferable outputs, platform design should be coupled with standardized quantitative and reporting frameworks (Figure 3). This framework summarizes commonly used enhancement metrics, functional response profiles, and minimum reporting standards, linking assay structure to downstream interpretation. Definition of baseline references, dilution ranges, peak and area-based metrics, and threshold criteria provides a shared analytical language consistent with comparative validation studies and inter-laboratory transfer efforts [16,17,21,38,40,44]. By embedding these metrics and reporting conventions within platform architecture, experimental design is aligned with reproducibility, cross-site harmonization, and translational relevance from the outset.

### 4.2. Immune Complex Formation, Viral Input Control, and Quality Assurance

ADE phenotypes are intrinsically concentration-dependent and therefore require a systematic evaluation across predefined antibody dilution ranges. Platform-based assays should incorporate standardized dilution series spanning neutralizing, transitional, and enhancing concentrations, with ranges adapted to expected antibody titers and study context. Figure 3A,B illustrate FcγR-mediated uptake and downstream functional readouts, including infection frequency and viral output. Immune complex formation conditions, including incubation time, temperature, and mixing procedures, should be harmonized within and across sites. Documentation of these parameters is essential for interpretability and reproducibility. Viral input standardization is central to platform stability. Viral stocks should be quantified using predefined primary metrics (e.g., PFU, FFU, or IU) and characterized for batch-to-batch variability, particle infectivity ratios, and storage stability. Where possible, reference virus lots or bridging panels should be incorporated to facilitate inter-laboratory harmonization. Freeze–thaw cycles, passage history, and preparation methods should be recorded systematically. Deviations from established specifications should trigger predefined review or recalibration procedures. Normalization and quality assurance procedures form the backbone of transferable ADE platforms. Each experimental run should incorporate virus-only baselines, positive control sera, and plate-level controls to enable intra- and inter-assay comparison. Acceptance thresholds for control performance should be established prospectively. Batch-tracking systems should link outputs to reagent lots, cell passages, and operator metadata. In multi-site studies, the centralized monitoring of QC metrics enables early identification of systematic drift and supports coordinated corrective action. The importance of rigorous normalization and reference controls has been emphasized in comparative and validation studies, particularly during inter-laboratory transfer [16,17,21,38,44].

### 4.3. Readout Integration, Quantitative Metrics, and Interpretive Frameworks

Primary readouts should be selected according to study objectives and may include infection frequency, reporter activity, viral RNA or antigen production, infectious progeny output, and immunological response markers. Platforms intended for translational or preparedness applications benefit from integrating complementary readouts that capture both entry-level and downstream biological consequences. When multiple readouts are employed, analytical pipelines should predefine prioritization and weighting schemes to avoid post hoc interpretation bias. Integrated analysis enhances robustness and supports mechanistic and predictive modeling. Functional ADE platforms should employ pre-specified quantitative metrics that summarize enhancement and neutralization behavior across dilution ranges. Commonly applied measures include peak enhancement values, enhancement area-under-curve indices, and threshold-based indicators. Selection of metrics should reflect study goals and anticipated downstream use. Interpretive frameworks should distinguish between neutralization-dominant, enhancement-dominant, and mixed-response profiles and should account for assay architecture and biological context. Reporting should emphasize comparative patterns rather than isolated point estimates. Functional readouts should not be interpreted in isolation but integrated with complementary immunological parameters, including binding profiles, cellular immunity, exposure history, and repertoire-level information where available. Quantitative metrics and interpretive frameworks (Figure 3C) provide standardized approaches for summarizing enhancement profiles across experimental conditions.

In this context, variation in target cell systems directly influences quantitative readouts and response classifications. To preserve interpretability across cellular models, functional outcomes should be mapped to standardized response profiles and reporting conventions, as outlined in Figure 3. Applying shared baseline definitions, enhancement metrics, and zone-based interpretations enables results obtained in different FcγR-expressing systems to be compared within a common analytical framework, despite underlying biological differences. This linkage between cellular architecture and standardized metrics is essential for cross-platform validation and translational interpretation.

### 4.4. Reporting Standards, Data Governance, and Translational Alignment

**Figure 4:**
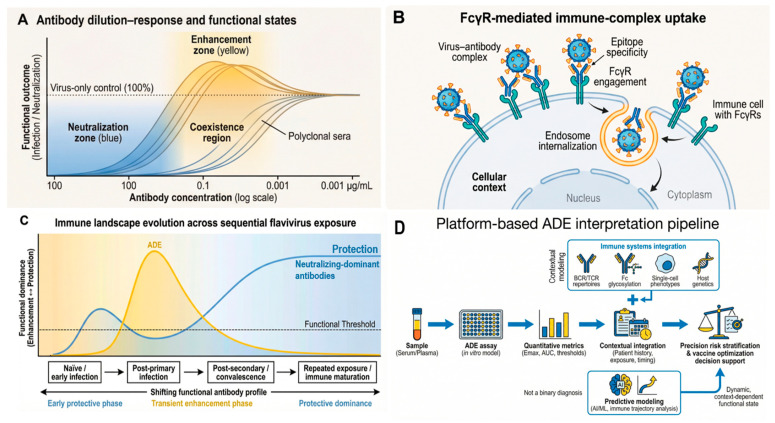
The functional interpretation of antibody-dependent enhancement as a dynamic immune state. A conceptual model illustrating how neutralization and enhancement emerge as interrelated functional outcomes across antibody concentration and immune-context gradients. Yellow shading denotes enhancement-prone (ADE) functional states, whereas blue shading denotes neutralization-dominant protective states. (**A**) Representative antibody dilution–response profiles demonstrating overlapping neutralization (blue) and enhancement (yellow) zones within polyclonal sera, including coexistence regions and context-dependent transitions. (**B**) Influence of epitope specificity, Fcγ receptor engagement, and cellular context on functional outcomes following immune complex uptake and endosomal processing. (**C**) Longitudinal evolution of functional antibody profiles following sequential flavivirus exposure. In immunologically naïve individuals, functional readouts remain near the baseline. Following primary infection, cross-reactive and subneutralizing antibodies generate a transient enhancement-prone profile. After secondary infection and immune maturation, profiles progressively shift toward neutralization-dominant states, reflecting increased affinity, breadth, and stoichiometric coverage. This trajectory illustrates that functional ADE signatures evolve dynamically over time rather than representing a fixed immunological status. (**D**) Platform-based interpretation pipeline integrating quantitative enhancement metrics with clinical and immunological context to support translational interpretation and precision risk stratification. This framework interfaces with emerging systems immunology and repertoire-based analytics, enabling integration of functional ADE metrics with BCR/TCR profiling, Fc glycosylation patterns, host genetic variation, and predictive modeling. Together, these panels emphasize that ADE represents a context-dependent, dynamic functional state rather than an intrinsic indicator of vaccine failure or adverse immunological risk.

To support reproducibility and secondary analysis, ADE studies adopting platform-based approaches should report, at a minimum: dilution ranges, baseline definitions, replicate structures, viral input specifications, target cell characterization procedures, and normalization methods. The materials and methods description should provide sufficient detail to enable independent replication and protocol transfer. Where applicable, contextual metadata—including cohort characteristics, vaccination or infection histories, and sampling intervals—should be integrated with functional outputs. The integration of these components into a structured analytical pipeline (Figure 4) enables the translation of experimental outputs into decision-relevant interpretations. Sustainable functional ADE platforms require coordinated data governance and documentation practices. Standardized data formats, version-controlled analytical pipelines, and curated reference datasets facilitate longitudinal analyses and future methodological upgrades. Consortium-based initiatives could support the establishment of governance structures for protocol revision, reference material renewal, and training dissemination. These mechanisms enable continuous platform evolution while preserving comparability with historical datasets. Platform-based ADE assessment is most impactful when aligned with clearly defined translational and preparedness goals, including vaccine safety profiling, immune landscape surveillance, and risk stratification in immunologically complex populations. Study designs should therefore incorporate decision-relevant outputs and stakeholder requirements from early stages. The integration with regulatory, funding, and public health frameworks enhances platform utility and supports incorporation into broader pandemic readiness infrastructures.

## 5. Discussion

Functional antibody-dependent enhancement (ADE) platforms have evolved from specialized mechanistic tools into core components of flavivirus immunology and vaccine evaluation. This transition reflects increasing recognition that conventional serological metrics alone are insufficient to capture functional immune outcomes in populations with complex exposure histories [5,6,7,20,21,23]. By integrating Fcγ receptor (FcγR) biology with standardized experimental workflows and quantitative metrics, platform-based approaches enable the structured, comparable assessment of antibody function across cohorts, interventions, and time points [12,16,17,19,20,21,22]. A central insight emerging from these systems is that enhancement and neutralization represent dynamic, concentration- and context-dependent functional states rather than fixed properties of antibody repertoires. As outlined in Section 2, these states are shaped by immune complex stoichiometry, FcγR engagement, cellular context, and viral characteristics [3,10,11,13,22,27]. Platform-based ADE assays, therefore, provide a means to resolve functional heterogeneity that cannot be captured by single-parameter measurements. This is particularly relevant in dengue-endemic regions, where sequential infections and cross-reactive immunity generate complex antibody landscapes that evolve over time [5,7,8,42,43].

Operational experience across multiple studies has further demonstrated that reproducibility in ADE assessment depends not only on biological factors but also on methodological governance. Variability in viral input, target cell conditions, and normalization procedures can significantly influence quantitative outputs if not systematically controlled [16,17,21,38,44]. Embedding quality control frameworks, reference materials, and standardized reporting structures within platform design enables a conversion of experimental measurements into interpretable, decision-relevant datasets. These features are essential for inter-laboratory comparability and for the integration of functional ADE assessment into broader translational and public health workflows.

At the same time, increasing integration with complementary technologies is expanding the analytical scope of ADE platforms. Coupling functional readouts with B cell receptor repertoire analysis, single-cell immune profiling, Fc glycosylation studies, and computational modeling provides a pathway toward a systems-level interpretation of immune function [7,8,42,52]. These approaches support the construction of immune landscape models linking antibody diversity, exposure history, and functional outcomes, with potential applications in vaccine design and population-level risk stratification. From a translational perspective, functional ADE platforms are increasingly positioned as tools for contextual interpretation rather than binary risk assessment. Enhancement signals should not be interpreted in isolation but evaluated alongside neutralization profiles, immunological parameters, and clinical metadata [8,10,13,16,18,30,31,33]. This integrated framework is particularly relevant for assessing vaccine responses, booster strategies, and cross-reactive immunity in heterogeneous populations [47,48,49].

However, despite their utility, functional antibody-dependent enhancement (ADE) platforms have important limitations that should be explicitly recognized. First, commonly used FcγR-expressing cell line systems, including FcγRIIa-expressing BHK models, recapitulate the partial biological complexity of primary human myeloid cells. Differences in receptor diversity, intracellular signaling networks, and innate immune responses may influence both viral replication dynamics and the magnitude of measured enhancement. As such, results obtained in these systems should be interpreted as controlled functional readouts rather than direct representations of in vivo cellular responses. Second, the relationship between in vitro ADE measurements and clinical disease severity remains incompletely defined. While enhancement phenotypes have been associated with specific immune contexts, current evidence indicates that disease outcomes are determined by multifactorial interactions involving viral load, host genetics, immune regulation, and prior exposure history. Functional ADE metrics therefore represent context-dependent immune states, rather than deterministic predictors of pathological outcomes, consistent with observations from the cohort and mechanistic studies [32,50,53,54,55]. Taken together, these considerations underscore that functional ADE platforms are best understood as structured immune assessment tools that support comparative and longitudinal analysis, rather than standalone indicators of clinical risk. Their interpretive value is maximized when integrated with complementary immunological, virological, and epidemiological data within a multi-parameter framework.

To date, the long-term application of FcγR-based functional platforms across multiple cohorts, vaccine trials, and epidemiological settings has demonstrated their durability and analytical value over more than a decade of deployment [10,11,12,16,17,18,19,20,21,22,23,24,25,26,27,44,45], supporting their role as core infrastructure for translational flavivirus immunology. However, several limitations warrant consideration. No single assay architecture can fully recapitulate the complexity of in vivo immune–virus interactions, and functional readouts remain sensitive to target cell selection and viral input properties. Moreover, large-scale implementation requires sustained investment in training, reference materials, and data governance. Addressing these challenges will require coordinated efforts among academic institutions, funding agencies, and public health stakeholders to maintain platform continuity and methodological rigor. Looking forward, the continued maturation of functional ADE platforms will depend on three interrelated priorities: (i) consolidation of reporting standards and reference frameworks; (ii) integration with multi-omic and computational modeling pipelines; and (iii) alignment with regulatory and policy processes governing vaccine evaluation and surveillance. Progress in these areas will transform ADE assessment from a specialized analytical activity into a routine component of translational immunology infrastructure. An important implication of platform-based ADE assessment is that enhancement should not be interpreted as an intrinsic marker of vaccine failure or immunological hazard. Rather, ADE reflects a natural functional consequence of polyclonal antibody responses operating within Fc-dependent biological systems, in which neutralizing and enhancing activities coexist across overlapping concentration ranges in a polyclonal antibody background, which is detected as the sum of the total antibody activity. Protective immunity and enhancement are therefore not opposing phenomena, but interrelated functional dimensions shaped by antibody concentration, epitope specificity, Fcγ receptor engagement, and host cellular context [3,10,11,13,22,27].

## 6. Conclusions

From this perspective, antibody-dependent enhancement should be understood as a measurable component of the broader immune landscape rather than as an intrinsic marker of pathological risk. The objective of the vaccine design is therefore not the elimination of enhancement-prone activity but the rational shaping of antibody responses toward dominant protective function across physiologically relevant conditions. Functional ADE platforms provide a quantitative and comparative framework for evaluating this balance and for tracking how immune states evolve following infection, vaccination, or boosting. Their continued refinement and integration with multi-parameter immunological data will be essential for advancing translational interpretation and supporting evidence-based decision-making in flavivirus research and beyond.

## Data Availability

No new data were created or analyzed in this study.

## Abbreviations

The following abbreviations are used in this manuscript:

ADE	Antibody-Dependent Enhancement
AUC	Area Under the Curve
BCR	B-Cell Receptor
DENV	Dengue Virus
ECBS	WHO Expert Committee on Biological Standardization
E_max	Maximum Enhancement
E_AUC	Enhancement Area Under the Curve
FcγR	Fc Gamma Receptor
FFU	Focus-Forming Unit
FRNT	Focus Reduction Neutralization Test
HLA	Human Leukocyte Antigen
ICS	Intracellular Cytokine Staining
IFN-γ	Interferon Gamma
IgG	Immunoglobulin G
IU	Infectious Unit
JEV	Japanese Encephalitis Virus
MNT	Microneutralization Test
PBMC	Peripheral Blood Mononuclear Cell
PFU	Plaque-Forming Unit
PRNT	Plaque Reduction Neutralization Test
QC	Quality Control
RT-PCR	Reverse Transcription PCR
SRIP	Single-Round Infectious Particle
TCR	T-Cell Receptor
